# Liver resection at the time of secondary cytoreduction in ovarian cancer^☆^

**DOI:** 10.1016/j.gore.2026.102022

**Published:** 2026-04-14

**Authors:** Christina Harlev, Lindsey Finch, Dennis S. Chi, William R. Jarnagin, Kevin Soares

**Affiliations:** a Memorial Sloan Kettering Cancer Center, Department of Surgery, Gynecology Service, New York, NY, USA; b Weill Cornell Medical College, Department of Obstetrics and Gynecology, New York, NY, USA; c Memorial Sloan Kettering Cancer Center, Department of Surgery, Hepatopancreatobiliary Service, New York, NY, USA

**Keywords:** Cytoreduction, Ovarian cancer, Hepatic resection

## Abstract

**Background:**

Hepatic involvement in recurrent ovarian cancer has historically been considered a relative contraindication to cytoreductive surgery. Advances in hepatobiliary surgery and multidisciplinary collaboration have expanded the role of liver resection (Farges et al., 2012, Fong et al., 2005, Hiatt et al., 1994, Curley and Glazer, 2025. This video demonstrates the technical considerations and feasibility of hepatic resection during secondary cytoreductive surgery for ovarian cancer.

**Case:**

A 61-year-old woman with early-stage ovarian cancer and a long disease-free interval developed an isolated hepatic recurrence. Imaging revealed a centrally necrotic mass in the right hepatic lobe with contiguous involvement of the right adrenal gland. Biopsy confirmed metastatic high-grade Müllerian carcinoma. Following systemic chemotherapy with radiographic response, the patient was selected for secondary cytoreductive surgery.

**Surgical technique:**

The procedure included partial hepatectomy of segments V and VI with en bloc right adrenalectomy and cholecystectomy. Small-volume pelvic disease was removed via low anterior rectal resection, achieving complete gross tumor resection. The video highlights hepatic mobilization, identification of key anatomic landmarks including the hepatoduodenal ligament, Glissonian pedicles, and hepatic venous structures, and parenchymal transection using the clamp-crush technique with vascular control via the Pringle maneuver. Intraoperative ultrasound confirmed vascular anatomy and perfusion of the remnant liver.

OutcomeEstimated blood loss was 450 mL with a Pringle time of 25 min. Pathology confirmed metastatic high-grade Müllerian adenocarcinoma with negative hepatic margins. The patient was discharged on postoperative day ten without major postoperative complications.

**Conclusion:**

Hepatic resection during secondary cytoreductive surgery for ovarian cancer is safe and feasible in carefully selected patients when performed by experienced multidisciplinary teams with detailed knowledge of hepatic anatomy.

## 1 Consent Statement

Informed consent was obtained from the patient for publication of this report and accompanying media.

## CRediT authorship contribution statement

**Christina Harlev:** Investigation, Visualization, Writing – original draft, Writing – review & editing. **Lindsey Finch:** Visualization, Writing – original draft, Writing – review & editing. **Dennis S. Chi:** Conceptualization, Supervision, Writing – review & editing. **William R. Jarnagin:** Methodology, Writing – review & editing. **Kevin Soares:** Methodology, Writing – review & editing.

## Funding

This work was supported in part by a Cancer Center Support Grant from the National Institutes of Health/National Cancer Institute (P30CA008748).

## Declaration of competing interest

The authors declare the following financial interests/personal relationships which may be considered as potential competing interests: C. Harlev reports funding from the Danish Cancer Society. L. Finch reports common stock in Johnson & Johnson. D. Chi declares royalties from McGraw Hill, Taylor and Francis, Wolter Kluwer; speaker fees from AstraZeneca; advisory board participation with Verthermia Acquio, Biom ‘Up; stock in Doximity (sold in 2024), BioNTech SE (sold in 2023). The remaining authors report no conflicts.
